# Evaluating the effects of protective ventilation on organ-specific cytokine production in porcine experimental postoperative sepsis

**DOI:** 10.1186/s12890-015-0052-9

**Published:** 2015-05-10

**Authors:** Jesper Sperber, Miklós Lipcsey, Anders Larsson, Anders Larsson, Jan Sjölin, Markus Castegren

**Affiliations:** Centre for Clinical Research Sörmland, Uppsala University, Uppsala, Sweden; Department of Medical Sciences, Infectious Diseases, Uppsala University, Uppsala, Sweden; Department of Surgical Sciences, Hedenstierna laboratory, Anaesthesiology and Intensive Care, Uppsala University, Uppsala, Sweden; Department of Medical Sciences, Biochemical structure and function, Uppsala University, Uppsala, Sweden; Department of Anaesthesiology and Intensive Care, Mälarsjukhuset Eskilstuna, Sweden

**Keywords:** Mechanical ventilation, Protective ventilation, Endotoxemia, Experimental sepsis, Porcine

## Abstract

**Background:**

Protective ventilation with lower tidal volume (V_T_) and higher positive end-expiratory pressure (PEEP) reduces the negative additive effects of mechanical ventilation during systemic inflammatory response syndrome. We hypothesised that protective ventilation during surgery would affect the organ-specific immune response in an experimental animal model of endotoxin-induced sepsis-like syndrome.

**Methods:**

30 pigs were laparotomised for 2 hours (h), after which a continuous endotoxin infusion was started at 0.25 micrograms × kg^−1^ × h^−1^ for 5 h. Catheters were placed in the carotid artery, hepatic vein, portal vein and jugular bulb. Animals were randomised to two protective ventilation groups (n = 10 each): one group was ventilated with V_T_ 6 mL × kg^−1^ during the whole experiment while the other group was ventilated during the surgical phase with V_T_ of 10 mL × kg^−1^. In both groups PEEP was 5 cmH_2_O during surgery and increased to 10 cmH_2_O at the start of endotoxin infusion. A control group (n = 10) was ventilated with V_T_ of 10 mL × kg^−1^ and PEEP 5 cm H_2_0 throughout the experiment. In four sample locations we a) simultaneously compared cytokine levels, b) studied the effect of protective ventilation initiated before and during endotoxemia and c) evaluated protective ventilation on organ-specific cytokine levels.

**Results:**

TNF-alpha levels were highest in the hepatic vein, IL-6 levels highest in the artery and jugular bulb and IL-10 levels lowest in the artery. Protective ventilation initiated before and during endotoxemia did not differ in organ-specific cytokine levels. Protective ventilation led to lower levels of TNF-alpha in the hepatic vein compared with the control group, whereas no significant differences were seen in the artery, portal vein or jugular bulb.

**Conclusions:**

Variation between organs in cytokine output was observed during experimental sepsis. We see no implication from cytokine levels for initiating protective ventilation before endotoxemia. However, during endotoxemia protective ventilation attenuates hepatic inflammatory cytokine output contributing to a reduced total inflammatory burden.

## Background

Biotrauma from mechanical ventilation comprises overextension of alveoli, cyclic atelectasis, activation of immune cells and spill of inflammatory mediators to the systemic circulation [1]. Protective ventilation (PV), i.e. the reduction of biotrauma by the use of small tidal volumes and appropriate positive end-expiratory pressure (PEEP), has reduced morbidity and mortality in clinical studies [2,3]. Experimentally, mechanical ventilation has been linked to increased susceptibility to inflammatory mediator induced lung damage by inducing Toll-like receptors [4]. The inflammatory response to infection and trauma can be quantified by concentrations of immune mediators in blood, such as cytokines secreted from activated immune cells. Organ damage from excessive inflammatory responses may be caused by systemic cytokine levels [5] but theoretically may also be linked to organ-specific cytokine production. However, data are scarce regarding the contribution of cytokines from individual organs to the systemic picture.

Arguably, the lack of knowledge in this area can possibly be a detriment to the development of tools to avoid organ dysfunction. As biomarkers, sustained elevated plasma levels of cytokines have been correlated with poor outcome in sepsis and trauma [5,6]. The mechanistic relation, complicated because of the pleiotropic function of cytokines, between the pro-inflammatory IL-6 and organ dysfunction has recently been established in an animal model [7]. These results suggest the possible use of cytokine levels not only as correlative biomarkers but also as clinical targets.

The study, set up to mimic a clinical setting with postoperative systemic inflammation, had three aims: to analyse whether organ-specific plasma levels of TNF-α, IL-6 and interleukin 10 (IL-10) differed from the corresponding arterial levels; to evaluate the effects of early protective ventilation initiated 2 hours (h) before endotoxemia compared with protective ventilation only during the endotoxemic period on extra-pulmonary organ-specific cytokine levels; and to analyse the effects of protective ventilation compared with medium high tidal volume ventilation on extra-pulmonary organ-specific cytokine levels.

## Methods

### Ethics statement

Thirty healthy pigs of both genders aged 9 to 12 weeks and sexually immature were included in the study. Until 1 h before the experiment, the animals had free access to food and water. Surgery was performed under balanced general anaesthesia and all efforts were made to minimise animal suffering. All animals were handled in accordance with the animal experimentation guidelines established by the Swedish Board of Agriculture. The study was approved by the Animal Ethics Board (Uppsala djurförsöksetiska nämnd, permit no. C250/11) in Uppsala, Sweden.

### Anaesthesia and surgical procedure

The experiment was set up to mimic a clinical scenario of postoperative sepsis, in which the impact of ventilator settings initiated before and after the experimental complication (endotoxemia) could be evaluated. The inflammatory response from the surgery was considered more important than the occurrence of an actual surgical intervention, such as colectomy, in this model. Therefore, the preparatory surgery (comprising skin incisions in the neck for tracheostomy and bilateral vessel access, a small laparotomy for bladder access and a larger laparotomy for access to the splenic vessels for catheterisation of the portal vein) had the additional task of generating a standardised systemic trauma response. The magnitude of response is presented in an earlier publication [8]. The samples for the present study were taken concomitantly with samples from an earlier experiment, which aimed to describe the impact of protective ventilation during endotoxemia on systemic inflammation, end organ damage and physiologic variables [8]. Data from the present study have not been previously published. The endotoxin dose has been validated in a previous publication together with detailed descriptions of the anaesthetic procedure, preparations and intensive care protocol [9]. In summary, premedicated animals were anaesthetised before tracheal intubation and thereafter mechanically ventilated throughout the experiment (Servo 900C or Servo i, Siemens Elema, Stockholm, Sweden). The start of mechanical ventilation marked the beginning of the experiment (denoted −2 h). Physiologic surveillance during the experiment included a 5 F catheter inserted 10 cm in a carotid artery branch, a central venous catheter, a 7 F Swan-Ganz catheter from the right side of the neck and a suprapubic urinary catheter. For the organ-specific sampling, in sequence, a 5 F catheter was placed in cranial direction via the left internal jugular vein to reach an approximated tip location of the jugular bulb, a 7 F Swan-Ganz catheter was placed in a hepatic vein via a pulmonary artery catheter introducer in the left external jugular vein and a 5 F catheter was placed in the vena porta via cannulation of the splenic hilus. The latter was accessed by a 20-cm long subcostal incision, blunt dissection and evisceration of the spleen. The spleen was relocated in the abdomen after catheter insertion but the wound was left open. The tip locations of the catheters in the hepatic and portal veins were confirmed by fluoroscopy and a total of 5 mL of iohexol contrast medium (Omnipaque™, GE Healthcare AB, Stockholm, Sweden). After surgical preparations were complete, a 30-min stabilisation period followed under which a Ringer’s acetate fluid bolus of 20 mL × kg^−1^ was given. Just before 0 h, the subcostal incision was closed with sutures and blood samples drawn from the different catheter locations.

### Protocol

**Figure 1:**
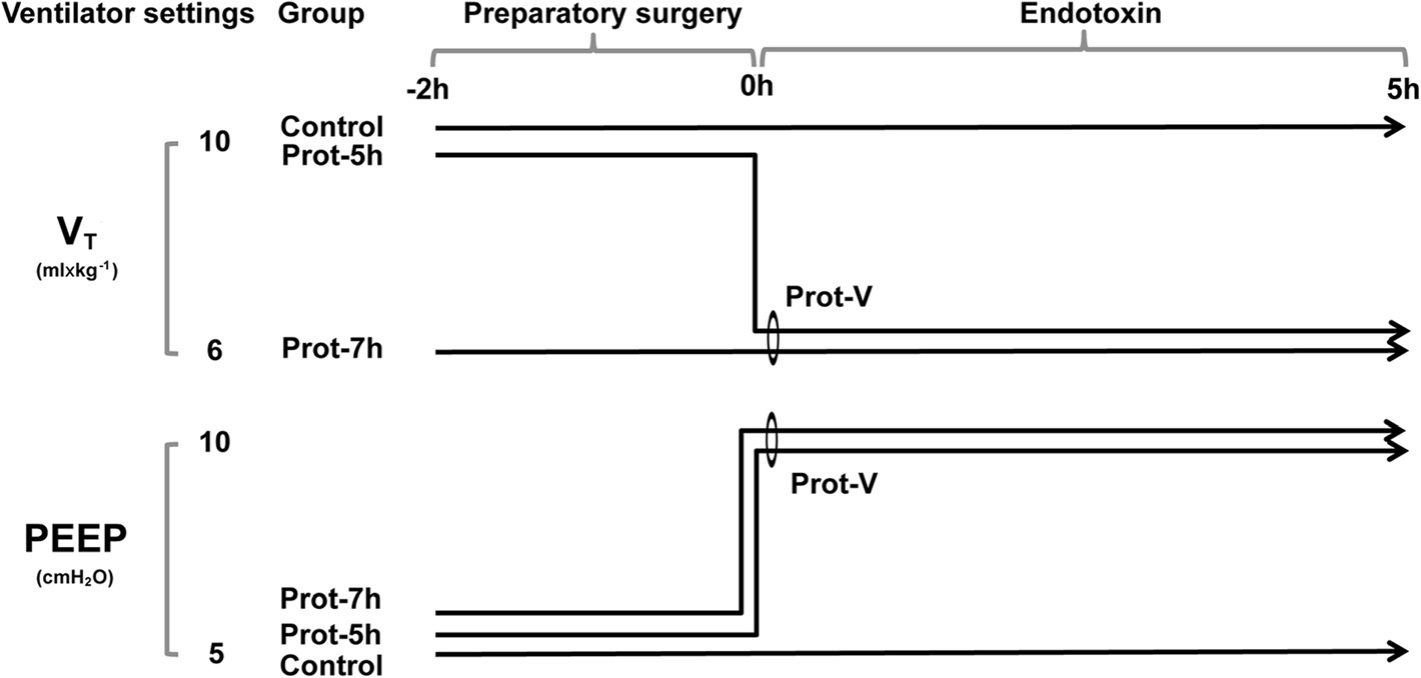
Overview of the experimental design. During the preparatory surgery (i.e. incisions for tracheostomy and for catheters in the neck and abdomen including temporary evisceration of the spleen), all three groups are n = 10. Prot-7 h was ventilated with V_T_ 6 mL × kg^−1^ and PEEP 5 cmH_2_O and Prot-5 h with V_T_ 10 mL × kg^−1^ and PEEP 5 cmH_2_O from −2 to 0 h. From 0 h, Prot-7 h and Prot-5 h were combined into one group, Prot-V (n = 20), and ventilated with V_T_ 6 mL × kg^−1^ and PEEP 10 cmH_2_O until the end of the experiment. The control group was ventilated with V_T_ 10 mL × kg^−1^ and PEEP 5 cmH_2_O for the entire experiment. From 0 h, an endotoxin infusion was given at a rate of 0.25 μg × kg^−1^ × h^−1^ until the end of the experiment.

Initial randomisation was done in blocks of 10 animals into three ventilation groups (Figure 1). The two protective ventilation groups, Prot-7 h and Prot-5 h, only differed in ventilator settings during the preparatory surgery phase between −2 and 0 h. The two ventilation groups were identical from 0 h until the end of the experiment. Prot-7 h (n = 10) had a V_T_ of 6 mL × kg^−1^ during the whole experiment, whereas Prot-5 h (n = 10) had a V_T_ during the surgery phase of 10 mL × kg^−1^ and 6 mL × kg^−1^ from 0 h until the end of the experiment. The control group (n = 10) had a V_T_ of 10 mL × kg^−1^ during the entire experiment. PEEP levels were 5 cm H_2_O in all groups (Prot-5 h, Prot-7 h and control) during the surgery phase, but were changed to 10 cm H_2_O in the two protective groups (Prot-7 h and Prot-5 h) at 0 h. In the control group the PEEP level was 5 cm H_2_O for the entire experiment. The initial respiratory frequency was 35 × min^−1^ in the Prot-7 h group with a V_T_ of 6 mL × kg^−1^ and 25 × min^−1^ in the two groups with a V_T_ of 10 mL × kg^−1^. Initial inspiratory fraction of oxygen (FiO_2_) was 0.3 in all groups. After completion of the experimental series, differences in levels of inflammatory cytokines at 0 h between the two protective ventilation groups were compared. Given that no trend towards a difference was noted between the two groups, they were combined (coded as Prot-V, n = 20) for analysis. An intravenous (i.v.) infusion of endotoxin (Escherichia coli, serotype 0111:B4) (Sigma Chemical Co., St Louis, MO, USA) of 0.25 μg × kg^−1^ × h^−1^ was started at 0 h. To avoid bacterial contamination all animals were given 20 mg × kg^−1^ of cefuroxime at 1 h.

### Interventions

Adjusting respiratory frequency in increments or decrements of 10% kept PaCO_2_ within pre-set limits between 5 and 5.5 kPa. FiO_2_ was increased or decreased by fixed steps based on arterial blood gas analysis according to the pre-set limits of PaO_2_ between 12 and 20 kPa. A drop in mean arterial blood pressure (MAP) to 50 mmHg and rise in mean pulmonary arterial pressure (MPAP) to 50 mmHg, as in previous studies using this model [10,11], were treated with fluid boluses of Ringers acetate 10 mL × kg^−1^ and additional epinephrine boluses of 0.1 mg during the first 90 min after endotoxin exposure. Fluid boluses and nor-epinephrine infusion in fixed levels were used for the same indication for the remainder of the experiment.

### Measurements

Blood samples were drawn from the artery, hepatic vein, portal vein and jugular bulb at 0, 1, 3 and 5 h to determine levels of inflammatory cytokines. The samples were centrifuged to retain plasma and immediately frozen for later analyses. Commercial porcine-specific sandwich enzyme-linked immunosorbent assay (ELISA) was used to determine TNF-α, IL-6 and IL-10 in plasma (DY690B (TNF-α) and DY686 (IL-6), R&D Systems, Minneapolis, MN, USA and KSC0102 (IL-10), Invitrogen, Camarillo, CA, USA). The lower detection limits in EDTA plasma were < 230 pg × mL^−1^ for TNF-α, < 60 pg × mL^−1^ for IL-6 and < 60 pg × mL^−1^ for IL-10. All ELISAs had intra-assay coefficients of variation (CV) of less than 5% and total CV of less than 10%.

### Endpoints, calculations and statistics

The power analysis was based on a detectable difference of 15% of TNF-α in systemic plasma at the experimental endpoint, an alpha error of 0.05, a power of 0.8 and a SD of 10%. Performed in a previous similar study design, the power analysis yielded six evaluable animals in each group [10]. However, the groups were expanded to 10 animals in the current experiment to increase the possibility of finding differences in organ-specific locations where we had no previous experience on cytokine levels. TNF-α, IL-6 and IL-10 concentrations were log-normally distributed and hence logarithmically transformed for statistical analysis. The dynamic progress of cytokine levels was evaluated during the whole experiment because morbidity in patients with sepsis has been correlated to the area under the curve rather than to the peak levels [5]. Thus, survival until the experimental endpoint was required and animals that died before the experimental endpoint were excluded and replaced. The group effect in the analysis of variance (ANOVA) for repeated measures was used in all outcome statistical analyses. The rational for the two protective ventilation groups, i.e. Prot-7 h from the start of the experiment (−2 h) and Prot-5 h from the time of endotoxin exposure (0 h), was to analyse whether an effect of early protective ventilation was present. To optimise the number of animals needed for the study the two groups would be merged into a single protective ventilation group from 0 h (n = 20) if no trend was evident towards a difference in organ-specific cytokine levels (TNF-α, IL-6 or IL-10 in v. hepatica, v. porta or the jugular bulb). The cut-off value for what was considered a trend towards a difference was defined as a p-value of less than 0.5, calculated by one-way ANOVA tests at 0 h. Statistica™ (Statsoft, Tulsa, OK) was used in the statistical calculations and for the control of relevant assumptions. Data are presented as mean values ± standard deviation (SD), unless otherwise stated. A p-value <0.05 was considered significant.

## Results

The 30 animals had a weight of 25.8 ± 1.5 kg. Two animals from the Prot-5 h group died in association with pulmonary hypertension induced by endotoxemia [12] and hence replaced by other animals. All animals developed symptoms of severe systemic inflammation after the start of endotoxin infusion. Circulatory deterioration was manifested by an increase in MPAP and decreases in cardiac index (CI) and MAP. Fluid boluses were given to 14 animals in median 20 mL × kg^−1^ (range 10 – 40), single epinephrine boluses were given to 13 animals, nor-epinephrine infusion was started in 3 animals and progressive increments of inspired oxygen fraction (FiO_2_) were performed in 13 animals. Hypoperfusion was manifested by a slight rise in arterial lactate. Organ dysfunction towards the end of the experiment was evidenced by reductions in arterial oxygen tension/inspired oxygen fraction (PaO_2_/FiO_2_) and in left ventricular stroke work index (LVSWI). A summary for all experimental animals (n = 30) is given in Table 1 to appreciate the model. However, detailed accounts of ventilator settings, physiologic variables and organ dysfunction variables on the group level have appeared elsewhere [8].

**Table 1:** **Physiological and laboratory parameters reflecting circulation, hypoperfusion and organ dysfunction**

**Time**	**MAP**	**CI**	**MPAP**	**Lactate**	**PaO**_**2**_ **/FiO**_**2**_	**LVSWI**
**(h)**	**(mmHg)**	**(Lxmin** ^**−1**^ **× m** ^**−2**^ **)**	**(mmHg)**	**(mmol × L** ^**−1**^ **)**	**(mmHg)**	**(gmxm** ^**−2**^ **× beat)**
**0**	91 ± 10	2.7 ± 0.5	27 ± 9	2.1(1.8/2.5)	476 ± 63	33 ± 9
**1**	88 ± 16	2.0 ± 0.5	42 ± 9	2.6(2.0/3.5)	397 ± 105	24 ± 10
**2**	86 ± 19	2.2 ± 0.4	38 ± 8	**-**	394 ± 101	24 ± 12
**3**	80 ± 23	1.9 ± 0.5	40 ± 5	3.0(2.6/3.6)	345 ± 115	18 ± 9
**4**	71 ± 17	2.0 ± 0.5	37 ± 5	**-**	315 ± 110	15 ± 6
**5**	65 ± 14	2.0 ± 0.5	35 ± 7	2.3(1.6/2.9)	319 ± 156	14 ± 6

Values are for all animals in the experiment (n = 30) from 0 to 5 hours. All values are mean ± SD, except lactate values which are median (range). MAP = mean arterial pressure, CI = cardiac index, MPAP = mean pulmonary arterial pressure, PaO_2_/FiO_2_ = arterial oxygen tension/inspired oxygen fraction, LVSWI = left ventricular stroke work index.

### Comparison between cytokine levels in different sample locations

**Figure 2:**
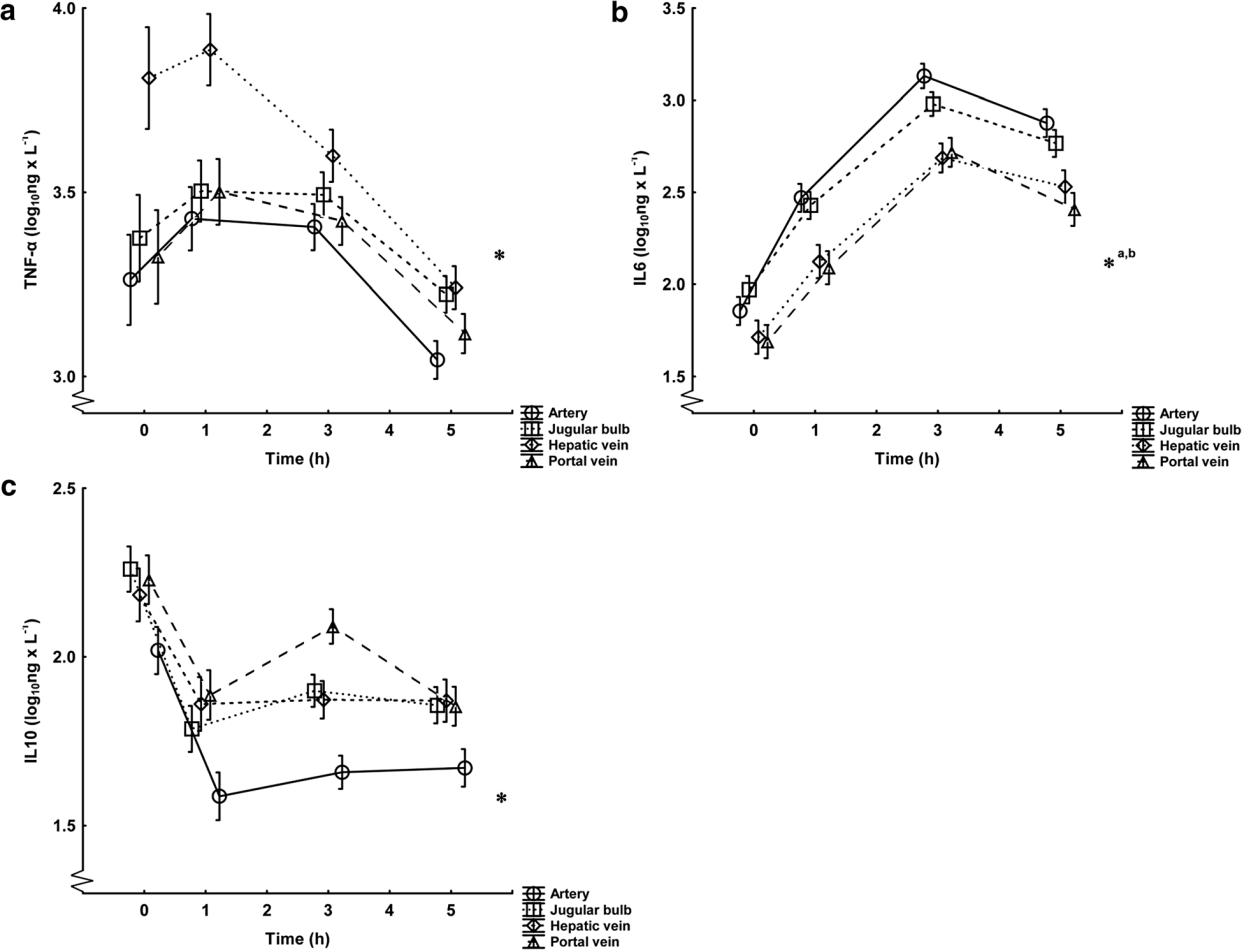
TNF-α, IL-6, and IL-10 levels from different sample locations. Values are logarithmically transformed (mean ± SD) and reflect all animals in the experiment (n = 30) from 0 to 5 hours. ANOVA for repeated measures, comparing individual sample locations over the whole experiment: **a)** TNF-α, *denotes the hepatic vein compared with the artery (p < 0.001), jugular bulb (p < 0.001) and portal vein (p < 0.001). **b)** IL-6, *^a^denotes the artery compared with the hepatic vein (p < 0.001) and portal vein (p < 0.001), *^b^denotes the jugular bulb compared with the hepatic vein (p < 0.01) and portal vein (p < 0.01). **c)** IL-10, *denotes the artery compared with the hepatic vein (p < 0.001), portal vein (p < 0.001) and jugular bulb (p < 0.001).

The levels of cytokines from the four plasma sample locations are presented in Figure 2a-c. TNF-α levels were higher in the hepatic vein than in the artery, jugular bulb and portal vein. IL-6 levels were higher in the artery and jugular bulb than in the portal and hepatic veins. No difference was found between IL-6 levels in the jugular bulb and the artery; nor were there differences between the portal and hepatic veins. IL-10 levels were numerically, but not significantly, higher in the portal vein compared with the jugular bulb and hepatic vein. IL-10 levels were lower in the artery than in the portal vein, hepatic vein and jugular bulb.

### Comparison between protective ventilation initiated before and during endotoxin on organ-specific cytokine levels

Levels of TNF-α, IL-6 and IL-10 in hepatic, portal and jugular bulb veins did not differ when the values at 0 h in group Prot-7 h were compared with those in group Prot-5 h (Table 2). Because no individual p-value was less than 0.5, the two groups were combined in the following analyses into the Prot-V group, according to the statistical plan. Additionally, to rule out late effects, a repeated measures ANOVA was performed between Prot-7 h and Prot-5 h groups from 0 to 5 h. No significant differences were found (data not shown).

**Table 2:** **Comparisons between Prot-7 h and Prot-5 h at 0 hours for TNF-α, IL-6 and IL-10**

**Cytokine**	**Location**	**Group**		**P-value**
		**Prot-7 h**	**Prot-5 h**	
**TNF α**	**Hepatic vein**	3.7 ± 0.7	4.0 ± 0.7	0.74
	**Portal vein**	3.4 ± 0.7	3.2 ± 0.7	0.80
	**Jugular bulb**	3.4 ± 0.6	3.3 ± 0.6	0.85
**IL 6**	**Hepatic vein**	1.8 ± 0.4	1.7 ± 0.5	0.53
	**Portal vein**	1.7 ± 0.4	1.5 ± 0.8	0.93
	**Jugular bulb**	2.0 ± 0.3	1.9 ± 0.4	0.79
**IL 10**	**Hepatic vein**	2.1 ± 0.3	2.3 ± 0.4	0.90
	**Portal vein**	2.2 ± 0.4	2.4 ± 0.4	0.54
	**Jugular bulb**	2.2 ± 0.4	2.3 ± 0.4	0.60

Log_10_ ng × L^−1^ (mean ± SD) at 0 h, p-values refer to one-way ANOVAs between Prot-7 h and Prot-5 h for each organ-specific sample location.

### Comparison between protective ventilation and controls on organ-specific cytokine responses

**Figure 3:**
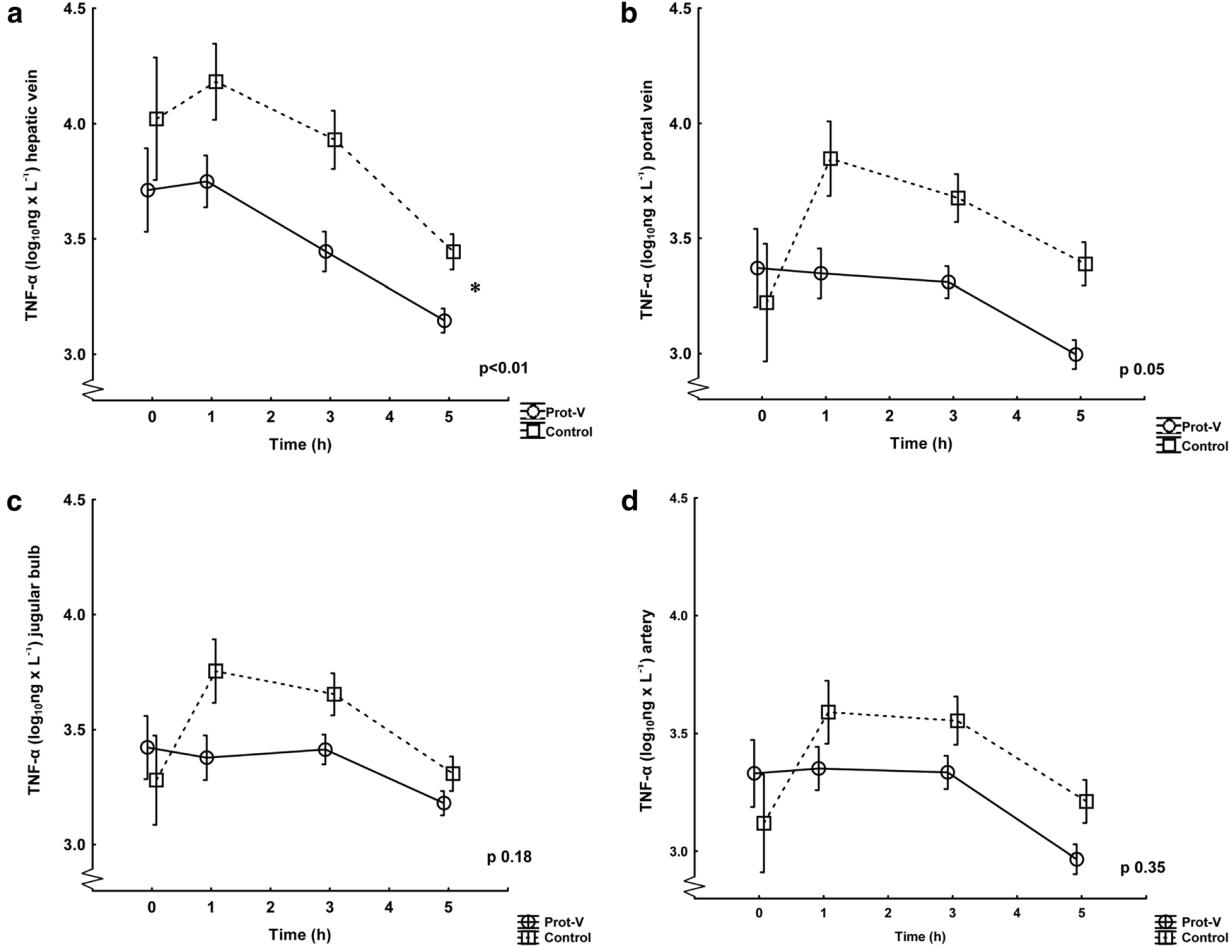
TNF-α levels in the ventilation groups Prot-V and Control. Values are logarithmically transformed (mean ± SD) from 0 to 5 hours. The Prot-V group included 20 pigs and the Control group 10. The p-values refer to ANOVA for repeated measures comparing the groups over the whole experiment. **a)** Hepatic vein, *denotes significance. **b)** Portal vein. **c)** Jugular bulb. **d)** Artery.

**Figure 4:**
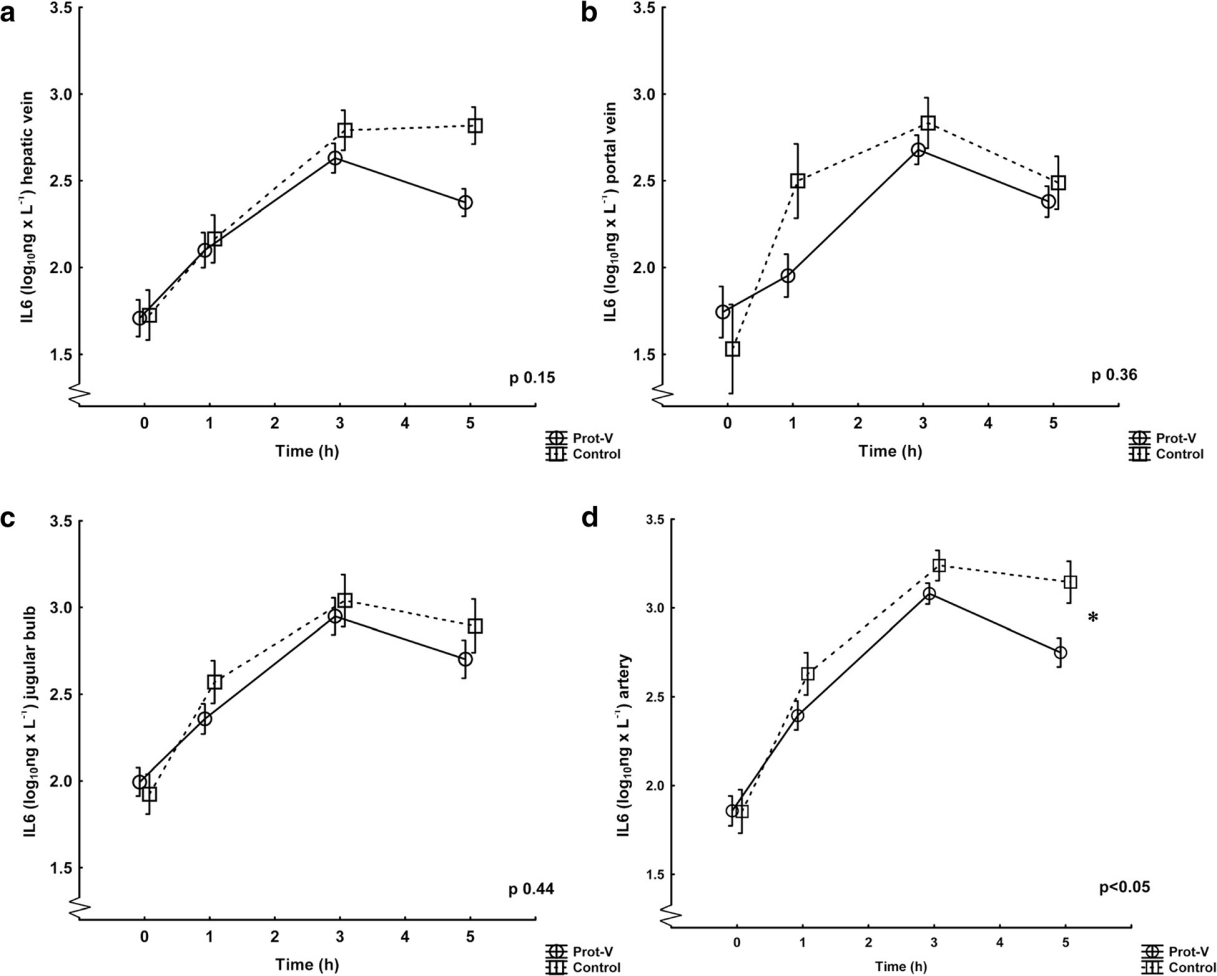
IL-6 levels in the ventilation groups Prot-V and Control. Values are logarithmically transformed (mean ± SD) from 0 to 5 hours. The Prot-V group included 20 pigs and the Control group 10. The p-values refer to ANOVA for repeated measures comparing the groups throughout the experiment. **a)** Hepatic vein. **b)** Portal vein. **c)** Jugular bulb. **d)** Artery, *denotes significance.

**Figure 5:**
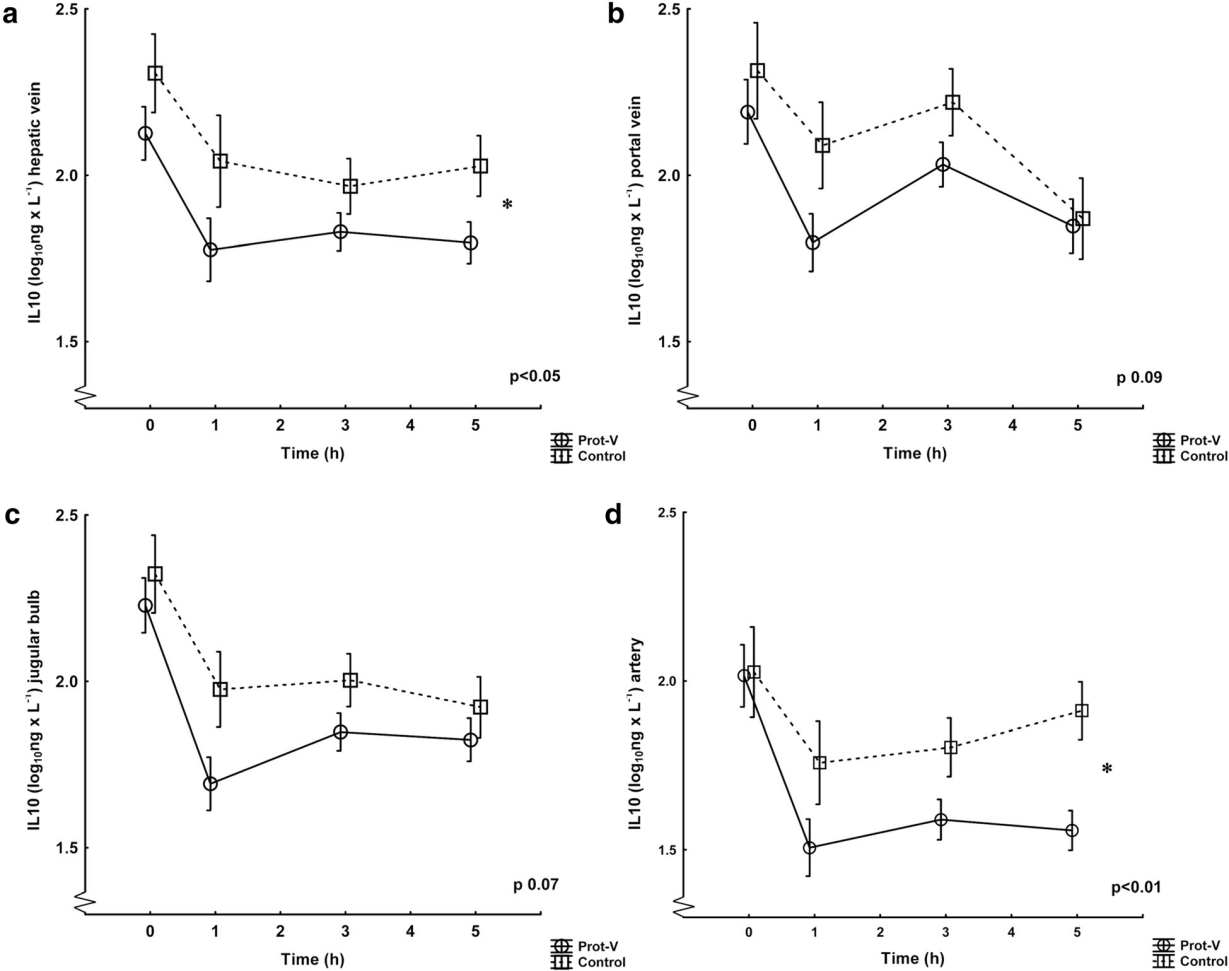
IL-10 levels in the ventilation groups Prot-V and Control. Values are logarithmically transformed (mean ± SD) from 0 to 5 hours. The Prot-V group included 20 pigs and the Control group 10. The p-values refer to ANOVA for repeated measures comparing the groups over the whole experiment. **a)** Hepatic vein, *denotes significance. **b)** Portal vein. **c)** Jugular bulb. **d)** Artery, *denotes significance.

The levels of cytokines from the different sample sites comparing the two ventilation groups (Prot-V and controls) are depicted in Figures 3, 4 and 5a-d. The hepatic vein showed significantly lower values during the whole experiment in the protective group for TNF-α and IL-10, whereas IL-6 values were numerically lower in the protective group at the end of the experiment (though not statistically significant). In the portal vein levels of all cytokines in the protective group were numerically lower, but only reached borderline significance for TNF-α. The jugular bulb showed lower numerical values in the Prot-V group for TNF-α and IL-10 but did not reach statistical significance over the whole experiment. No significant differences were seen for IL-6. In arterial samples no difference was noted between the ventilation groups for TNF-α; however, the control group had higher levels of IL-6 and IL-10.

## Discussion

The main findings were as follows: a) levels of cytokines differed significantly depending on sample location, b) protective ventilation initiated before endotoxin was not superior to that which was initiated concurrently with endotoxin and c) protective ventilation attenuated cytokines on an organ-specific level, most notably in the liver.

To our knowledge, no study has specifically aimed to compare cytokine levels from multiple sample sites in a large animal inflammatory model. The rational for the catheter placements in this study was based on cytokine responses previously described from these locations, i.e. the spleen and gut into the portal vein [13,14], liver [15], brain [16] and lungs [17]. Our results confirm studies that differentiate inflammatory responses from different organs and complicate the concept of “systemic” levels of cytokines. A recent study on sepsis patients compared plasma levels of TNF-α, IL-6 and IL-10 to immune-staining of freely circulating monocytes and leukocytes. Only small amounts of these cells indicated production, which supports the hypothesis that cytokine production primarily takes place in organ-resident cells, transmigrated cells or endothelia [18]. Additionally, a study on rats compared resident macrophages from lungs, peritoneum, liver and spleen and reported differences in cytokine production after *in vitro* endotoxin stimulation [19].

Two theoretical scenarios serve to discuss the observed differences in cytokines levels from the different sample locations. First, regarding location; what would it look like if one organ system were dominant in cytokine production from an inflammatory stimulus? Likely, levels of all cytokines would be the highest in efferent blood from this organ in comparison with other sample locations. Second, regarding specific cytokines; what would it look like if all organ systems acted uniformly in their reaction to an inflammatory stimulus? If so, the levels of different cytokines would be stacked in the same order independently of where the samples were taken, and only the magnitude of individual cytokine levels would differ between the locations. Our results, contrarily to the two proposed scenarios, strongly indicate that peak levels of different cytokines are located at different locations in the body, and that different organ systems preferentially produce certain cytokines. Most clearly, the levels of arterial cytokines – comparably the lowest in TNF-α and IL-10, but the highest in IL-6, illustrate this conclusion. The blood–brain barrier could potentially present a hinder to differentiate arterial levels from brain-derived levels in the jugular bulb, which makes these locations especially interesting to compare. The fact that IL10 levels significantly separate these two locations indicates that jugular bulb levels are not solely products of arterial levels.

Our study did not demonstrate any significant differences in cytokine expression between the two initial protectively ventilated groups (i.e. Prot-7 h and Prot-5 h) during surgery between −2 h and 0 h. The distinct reaction to surgery seen at 0 h in TNF-α and IL-10 would reasonably differentiate between two ventilation modes that were not equal in inflammatory attenuating ability. One reason for the failure to find differences could be the lack of power for this particular outcome measure. Another reason could be that the groups were only separated by tidal volume and not by PEEP during surgery. Hypothetically, if PEEP were the dominant factor in our combined intervention, a difference would not be expected in such a relatively short surgery time as 2 h.

The effects of protective ventilation could be seen in all the sample locations in this experiment. Although not reaching significant differences for all cytokines, all locations displayed lower absolute values in the protectively ventilated group as compared with the control group. The relatively small difference in tidal volume between the groups is unlikely to lead to such rapid and uniform repression of the inflammatory response from different organs. The adverse effect from the larger tidal volume would come from a massive alveolar over-stretch mechanism, and in this regard even the control group had clinically moderate tidal volumes. More likely, the cause would be the combined intervention or the PEEP level singularly. Interestingly, our observations suggest a general attenuation of inflammation induced by small differences in PEEP and tidal volume, but with a differential impact on organs. The possible mechanistic connection between mechanical ventilation and central neurogenic suppression of systemic inflammation is a highly promising research line [20].

We recognise limitations of our study design. First, we use the term “protective ventilation”, although the concept of PV is not well defined, but rather the absence of adding iatrogenic harm from mechanical ventilation. Previous landmark studies have indeed had a more thorough approach to establishing adequate PEEP levels based on titration and ventilator measurements [3]. Our intention is to reduce iatrogenic harm using small changes in ventilator settings, which motivates use of the terminology. Our approach to the concept of PV has been strictly practical and additionally seen from an operating room perspective. Therefore, we chose ventilator settings that would commonly be encountered clinically for both the intervention and control group [21]. On the same grounds, we omitted groups with zero PEEP and larger V_T_ commonly used to induce acute lung injury in experimental studies. Theoretically, if data increasingly support early protective ventilation in major surgery as a means to dampen the pro-inflammatory response, it would be easier to apply changes to the clinic if the changes were small and simple rather than large and complex. Second, our two-hit model was not designed to separate influences on cytokine levels of the two inflammatory stimuli. Therefore, we cannot differentiate the impact from PV on the separate stimuli. However, we consider the similarity to a clinical counterpart as a design strength that overbalances any weakness. Third, our results are obviously model-specific. Experiments have shown that the TNF-α responses in blood samples differ markedly between i.v. endotoxin and bacterial peritonitis, even though the models had the same lethality [22]. It might be argued that a bacterial peritonitis model would be more appropriate to simulate the septic complication related to major surgery. However, we chose our model for its ability to provide the same postoperative inflammatory stimulus to all animals and to be able to evaluate our intervention more effectively. Fourth, no flow measurement devices were placed in organ-specific locations. Thus, we were not able to relate measured concentrations of cytokines to flow. Accordingly, we cannot affirm with certainty that the significantly higher level of TNF-α observed in the hepatic vein is a true reflection of hepatic production. We would argue, however, that organ production of cytokines is the determining factor for cytokine counts at different locations [18]. If blood flow, on the other hand, were the dominant factor, there would probably be a more uniform picture in which all measured cytokines had the highest value in one location and the lowest in another. The cytokine levels from different organ locations in our study disconfirm this hypothesis.

The clinical implication of this study stems primarily from the finding that protective ventilation affects the TNF-α levels of the hepatic efferent circulation, which have previously been correlated with hepatocellular dysfunction and severity of adult respiratory distress syndrome (ARDS) [23,24]. The attenuation induced by protective ventilation on hepatic TNF-α production would therefore affect these areas in a clinically beneficial way and further favour the use of protective ventilation regimes outside of the ARDS domains. The chosen animal model in our study bridges the results from experiments performed on smaller animals [25,26], largely because humans share certain physiological and anatomical similarities with pigs but not with mice, rodents and rabbits [27].

## Conclusions

Cytokine output is differential between organs during experimental sepsis. We see no clinical implication from cytokine levels in this model for initiating protective ventilation before endotoxemia. However, during endotoxemia protective ventilation will attenuate hepatic inflammatory cytokine output and consequently reduce the total pro-inflammatory burden.

## Abbreviations

ANOVA: Analysis of variance
ARDS: Adult respiratory distress syndrome
CI: Cardiac index
cmH_2_O: Centimetre of water
CV: Coefficient of variation
ELISA: Enzyme-linked immunosorbent assay
F: French
FiO_2_: Inspiratory fraction of oxygen
H: Hour
i.e.: Id est
i.v.: Intravenous
IL-10: Interleukin 10
IL-6: Interleukin 6
Kg: Kilogram
kPa: Kilo Pascal
LVSWI: Left ventricular stroke work index
MAP: Mean arterial pressure
μg: Microgram
mg: Milligram
mL: Millilitre
mmHg: Millimetres of mercury
MPAP: Mean pulmonary arterial pressure
n: Number
PaCO_2_: Arterial tension of carbon dioxide
PaO_2_: Arterial tension of oxygen
PaO_2_/FiO_2_: Arterial oxygen tension/inspired oxygen fraction
PEEP: Positive end expiratory pressure
PV: Protective ventilation
SD: Standard deviation
TNF-α: Tumour necrosis factor alpha
v.: Vena
V_T_: Tidal volume

## References

[1] Tremblay LN, Slutsky AS (1998). Ventilator-induced injury: from barotrauma to biotrauma. Proc Assoc Am Physicians.

[2] Determann RM, Royakkers A, Wolthuis EK, Vlaar AP, Choi G, Paulus F (2010). Ventilation with lower tidal volumes as compared with conventional tidal volumes for patients without acute lung injury: a preventive randomized controlled trial. Crit Care.

[3] The Acute Respiratory Distress Syndrome Network (2000). Ventilation with lower tidal volumes as compared with traditional tidal volumes for acute lung injury and the acute respiratory distress syndrome. N Engl J Med.

[4] Villar J, Cabrera NE, Casula M, Flores C, Valladares F, Díaz-Flores L (2010). Mechanical ventilation modulates TLR4 and IRAK-3 in a non-infectious, ventilator-induced lung injury model. Respir Res.

[5] Pinsky MR, Vincent JL, Deviere J, Alegre M, Kahn RJ, Dupont E (1993). Serum cytokine levels in human septic shock. Relation to multiple-system organ failure and mortality. Chest.

[6] Gebhard F, Pfetsch H, Steinbach G, Strecker W, Kinzl L, Brückner UB (2000). Is interleukin 6 an early marker of injury severity following major trauma in humans?. Arch Surg Chic Ill 1960.

[7] Zhang Y, Zhang J, Korff S, Ayoob F, Vodovotz Y, Billiar TR (2014). Delayed neutralization of interleukin 6 reduces organ injury, selectively suppresses inflammatory mediator, and partially normalizes immune dysfunction following trauma and hemorrhagic shock. Shock Augusta Ga.

[8] Sperber J, Lipcsey M, Larsson A, Larsson A, Sjölin J, Castegren M (2013). Lung protective ventilation induces immunotolerance and nitric oxide metabolites in porcine experimental postoperative sepsis. PLoS ONE.

[9] Lipcsey M, Larsson A, Eriksson MB, Sjölin J (2008). Effect of the administration rate on the biological responses to a fixed dose of endotoxin in the anesthetized pig. Shock Augusta Ga.

[10] Carlsson M, Lipcsey M, Larsson A, Tano E, Rubertsson S, Eriksson M (2009). Inflammatory and circulatory effects of the reduction of endotoxin concentration in established porcine endotoxemic shock–a model of endotoxin elimination. Crit Care Med.

[11] Castegren M, Lipcsey M, Söderberg E, Skorup P, Eriksson M, Larsson A (2012). Differences in organ dysfunction in endotoxin-tolerant pigs under intensive care exposed to a second hit of endotoxin. Shock Augusta Ga.

[12] Schmidhammer R, Wassermann E, Germann P, Redl H, Ullrich R (2006). Infusion of increasing doses of endotoxin induces progressive acute lung injury but prevents early pulmonary hypertension in pigs. Shock Augusta Ga.

[13] Berg RD (1999). Bacterial translocation from the gastrointestinal tract. Adv Exp Med Biol.

[14] Huston JM, Ochani M, Rosas-Ballina M, Liao H, Ochani K, Pavlov VA (2006). Splenectomy inactivates the cholinergic antiinflammatory pathway during lethal endotoxemia and polymicrobial sepsis. J Exp Med.

[15] Ayala A, O’Neill PJ, Uebele SA, Herdon CD, Chaudry IH (1997). Mechanism of splenic immunosuppression during sepsis: key role of Kupffer cell mediators. J Trauma.

[16] Banks WA (2005). Blood–brain barrier transport of cytokines: a mechanism for neuropathology. Curr Pharm Des.

[17] Bhatia M, Zemans RL, Jeyaseelan S (2012). Role of chemokines in the pathogenesis of acute lung injury. Am J Respir Cell Mol Biol.

[18] Gille-Johnson P, Smedman C, Gudmundsdotter L, Somell A, Nihlmark K, Paulie S (2012). Circulating Monocytes Are Not the Major Source of Plasma Cytokines in Patients With Sepsis: Shock.

[19] Ogle CK, Wu JZ, Mao X, Szczur K, Alexander JW, Ogle JD (1994). Heterogeneity of Kupffer cells and splenic, alveolar, and peritoneal macrophages for the production of TNF, IL-1, and IL-6. Inflammation.

[20] Quilez ME, Fuster G, Villar J, Flores C, Martí-Sistac O, Blanch L (2011). Injurious mechanical ventilation affects neuronal activation in ventilated rats. Crit Care Lond Engl.

[21] Karalapillai D, Weinberg L, Galtieri J, Glassford N, Eastwood G, Darvall J (2014). Current ventilation practice during general anaesthesia: a prospective audit in Melbourne. Australia BMC Anesthesiol.

[22] Bagby GJ, Plessala KJ, Wilson LA, Thompson JJ, Nelson S (1991). Divergent efficacy of antibody to tumor necrosis factor-alpha in intravascular and peritonitis models of sepsis. J Infect Dis.

[23] Wang P, Ba ZF, Chaudry IH (1995). Hepatocellular dysfunction occurs earlier than the onset of hyperdynamic circulation during sepsis. Shock Augusta Ga.

[24] Callery MP, Kamei T, Mangino MJ, Flye MW (1991). Organ interactions in sepsis. Host defense and the hepatic-pulmonary macrophage axis. Arch Surg Chic Ill 1960.

[25] Imai Y, Parodo J, Kajikawa O, de Perrot M, Fischer S, Edwards V (2003). Injurious mechanical ventilation and end-organ epithelial cell apoptosis and organ dysfunction in an experimental model of acute respiratory distress syndrome. JAMA J Am Med Assoc.

[26] Brégeon F, Delpierre S, Chetaille B, Kajikawa O, Martin TR, Autillo-Touati A (2005). Mechanical ventilation affects lung function and cytokine production in an experimental model of endotoxemia. Anesthesiology.

[27] Parker SJ, Watkins PE (2001). Experimental models of gram-negative sepsis. Br J Surg.

